# Impact of the SARS-CoV-2 Pandemic on the Prevalence and Incidence of Enteric Protozoa in a Spanish Tertiary-Care Hospital and a Referral Center for Tropical Diseases, 2019–2023

**DOI:** 10.3390/medsci13010023

**Published:** 2025-03-01

**Authors:** Alfredo Maldonado-Barrueco, Fernando de la Calle-Prieto, Marta Díaz-Menéndez, Marta Arsuaga, Julio García-Rodríguez, Guillermo Ruiz-Carrascoso

**Affiliations:** 1 Clinical Microbiology and Parasitology Department, Hospital Universitario La Paz, 28046 Madrid, Spain; 2 National Referral Unit for Imported Diseases and International Health, High Level Isolation Unit, Hospital Universitario La Paz-Carlos III-CB, 14049 Madrid, Spain; 3 Centro de Investigación Biomédica en Red Enfermedades Infecciosas (CIBERINFEC), Instituto de Salud Carlos III, 28029 Madrid, Spain

**Keywords:** parasite, protozoa, gastroenteritis, *Blastocystis*, *Dientamoeba fragilis*, *Cryptosporidium*, *Giardia*, *Cyclospora cayetanensis*, *Entamoeba histolytica*, SARS-CoV-2, COVID-19

## Abstract

Objetive: The aim of this study was to describe the impact of non-pharmaceutical interventions (NPIs) against SARS-CoV-2 in patients with symptoms of enteric protozoa (EP), including *Blastocystis* spp., *Dientamoeba fragilis*, *Giardia lamblia*, *Cryptosporidium* spp., *Entamoeba histolytica*, and *Cyclospora cayetanensis*, in the overall population and in patients who were consulted at a National Referral Center for Imported Tropical Diseases (NRCITD patients) from a healthcare area in Madrid (Spain). Method: Data on patients with positive RT-PCR results for EP were collected. The periods analyzed were prepandemic (P0, 1 April 2019–31 March 2020), and the first (P1, 1 April 2020–31 March 2021), second (P2, 1 April 2021–31 March 2022), and third (P3, 1 April 2022–31 March 2023) pandemic years. We compared the prevalence, median age, absolute incidence (EP per 100,000 population of each period), and patient profile (NRCITD vs. non-NRCITD) during the study periods using Fisher’s test (*p* < 0.05) and the T-test (*p* < 0.001). Results: During P0, 24.8%, [95% CI: 23.9–25.6] of patients tested for EP RT-PCR were positive, 22.6% [95% CI: 21.5–23.7] were positive in P1, 20.4%, [95% CI: 19.5–21.3] were positive during P2, and 20% [95% CI: 19.2–20.9] of patients tested during P3 were positive. During the study, there was no difference in the median ages. The prevalence and absolute incidence of EP showed a decreasing trend during the pandemic for the NRCITD and non-NRCITD patients (*p* < 0.05). Conclusion: *Blastocystis* spp. and *D. fragilis* showed a lower decrease in prevalence during P1 (*p* > 0.05) due to the higher detection of colonized patients during the SARS-CoV-2 pandemic. However, *G. lamblia* and *Cryptosporidium* spp. showed the highest decrease in prevalence and absolute incidence during P2 (*p* < 0.05) because of the NPIs implemented during the SARS-CoV-2 pandemic. The NTRCID patients showed a higher prevalence of *Blastocystis* spp. than the non-NTRCID patients during every period studied (*p* < 0.001). *E. histolytica* and *C. cayetanensis* showed a homogeneous trend.

## 1. Introduction

On 14 March 2020, a state of alarm was declared in Spain because of the pandemic caused by the SARS-CoV-2 virus [1]. The movement of all citizens of the country was restricted except for essential personnel. At that time, strict limits on home visits and socializing in public places were established. National travel was suspended because of Spanish government regulations [1]. In addition, air traffic and international trips were reduced to one-off trips only [2]. Non-pharmaceutical interventions (NPIs) such as social distancing, sanitary hygiene measures, hand washing, the use of hydroalcoholic gels, and universal mask-wearing were made mandatory in all types of indoor and outdoor environments. In addition, there was the complete closure of recreational water sports facilities. These measures were gradually relaxed in Spain during the SARS-CoV-2 pandemic until the official declaration of the end of the pandemic by World Health Organization on 5 May 2023 [3]. We previously published a significant decrease in the prevalence and incidence of viral gastrointestinal illness and, to a lesser extent, a decrease in bacterial gastroenteritis illnesses during the SARS-CoV-2 pandemic in our health area [4,5]. However, the SARS-CoV-2 pandemic implied an increase in *Clostridioides difficile* healthcare facility-onset infection in our tertiary-care hospital [6].

The aims of this study were to evaluate the prevalence, absolute incidence (AI), temporal trends, and patient profile with enteric protozoa (EP) including *Blastocystis* spp., *Dientamoeba fragilis*, *Cryptosporidium* spp., *Giardia lamblia*, *Cyclospora cayetanensis*, and *Entamoeba histolytica* in a tertiary-care hospital and a National Referral Center for Imported Tropical Diseases (NRCITD) unit in Madrid (Spain) during the previous year and the first three years of the SARS-CoV-2 pandemic. We hypothesized that there could have been an overall reduction in EP because of the NPIs implemented during the SARS-CoV-2 pandemic. We also hypothesized that the decrease in tourism could contribute significantly to the decrease in the prevalence and incidence of EP among patients attending NRCITD units. Finally, we questioned whether the impact of NPIs during the SARS-CoV-2 pandemic was as noticeable on EP as previously reported for viral and bacterial gastrointestinal illnesses [4,5].

## 2. Materials and Methods

### 2.1. Population Data Analysis

This study was performed at the Hospital Universitario La Paz-Carlos III (HULP), a 1300-bed tertiary care hospital located north of Madrid (Spain) during 2019–2023. This hospital provides healthcare to 531,836 inhabitants (update to 2023) and serves as a referral hospital for 23 outpatient clinics. The clinical microbiology department receives stool samples of patients with gastrointestinal symptoms for screening of EP from the emergency department, inpatients, and outpatients under the designation of the overall population. In addition, the HULP includes a National Referral Center for Imported Tropical Diseases (NRCITD) and Health of Carlos III. This unit provides medical care to adult travelers, immigrants, and visiting friends and relatives (VFR), including EP screening (under the designation of NRCITD patients).

### 2.2. Microbiological Analysis

Macroscopic examination of stool samples was performed in the clinical microbiology department. Because cycles of diarrhea and non-diarrhea are characteristic of EP, both diarrheal and non-diarrheal stools were analyzed. Although the 2024 IDSA guideline recommends only one stool specimen for the molecular diagnosis of gastrointestinal parasitic infections [7], because of the increase in the sensitivity and cost-effectiveness of molecular techniques, three samples collected on different non-consecutive days were analyzed by pooling three stool samples of each patient. The outpatients preserved the stools in cold storage (1–4 °C) until the three samples were obtained for delivery to the clinical microbiology department following the instructions provided by the physicians.

The suspicion of EP was confirmed, and the pathogens were identified by real–time polymerase chain reaction (RT-PCR) using the Allplex™ GI-Parasite Assay (Seegene^®^, Seoul, Republic of Korea), along with automated DNA extraction and PCR setup using a Microlab STARlet Liquid Handling robot (Hamilton^®^, Reno, NY, USA), according to the manufacturers’ instructions. The RT-PCR assay includes the following six EP: *Blastocystis* spp., *Dientamoeba fragilis*, *Giardia lamblia*, *Cryptosporidium* spp., *Entamoeba histolytica*, and *Cyclospora cayetanensis*. Positive and negative controls were included in the reactions of each run. *Blastocystis* spp. and *D. fragilis* were considered positive when the cycle threshold (Ct) value was ≤35 due to the uncertain pathogenicity of these protozoa [8]. Low Ct values are correlate with a higher pathogenicity of these EP [9]. Due to the small number of patients who tested positive (n < 10) for *Cyclospora cayetanensis* and *Entamoeba histolytica* detection during the period studied, these EP were not statistically analyzed.

### 2.3. Study Design Analysis

In a retrospective observational cohort study design, we collected demographic and analytical data of patients with a positive result for any EP (as confirmed by RT-PCR) from the hospital database and laboratory informatics systems of the overall population and NTRCID patients. The four time periods analyzed were the prepandemic period (P0), from 1 April 2019 to 31 March 2020; the first SARS-CoV-2 pandemic year (P1), from 1 April 2020 to 31 March 2021; the second pandemic year (P2), from 1 April 2021 to 31 March 2022; and the third pandemic year (P3), from 1 April 2022 to 31 March 2023. Only the first sample of each patient was registered for each period of time to obtain a more representative dataset. The exception to this rule applied to patients who had episodes of EP caused by two different enteric protozoa (even if these episodes occurred within the same time period). This retrospective study was approved by the ethics committee of the HULP, code PI-5700.

### 2.4. Subanalysis in Patients Attending a National Referral Center for Imported Tropical Diseases (NRCITD)

To study whether the NPIs applied during the SARS-CoV-2 pandemic had a higher impact on EP in the NRCITD than non-NRCITD patients, a subanalysis of patients ≥18 years old with gastroenteritis symptoms, previous travel, and that were attending the NRCITD unit for the first time was performed. The variation in the number of patients between periods, the overall prevalence of EP, and AI between periods of EP were calculated for NRCITD patients. We calculated whether there were significant differences in the prevalence between NRCITD vs. non-NRCITD patients within each period. In addition, a comparison of the prevalence was performed between the groups of NRCITD patients for each pandemic period (P1–P3) related to the prepandemic period (P0).

### 2.5. Statistical Analysis Study

The median age and interquartile range (IQR) for each EP during the four periods studied were calculated. The overall prevalence, prevalences for each EP, prevalences between NRCITD and non-NRCITD patients, and the rate of AI per 100,000 population during the three SARS-CoV-2 pandemic periods were analyzed using Fisher‘s exact test compared with the prepandemic period (P0). A *p*-value of less than *p* < 0.05 was considered statistically significant. A subanalysis was performed between NRCITD vs. non-NRCITD patients. A T-test was performed to compare the monthly EP prevalences between NRCITD vs. non-NRCITD patients within each period. A *p*-value of less than 0.001 was considered statistically significant. Statistical analysis was performed using R Studio version 4.2.1.

## 3. Results

### 3.1. Overall Prevalence, Incidence, and Age Analysis

The total number of patients screened for EP in the prepandemic year (P0) was 9686; for the first pandemic year (P1), it was 5953; and for the second pandemic year (P2), it was 7749 (details in Table 1).

In this study, the overall EP prevalence and AI showed a decreasing trend that was statistically significant between the prepandemic and pandemic periods (*p* < 0.05) (details in Table 1 and Table 2).

**Figure 1:**
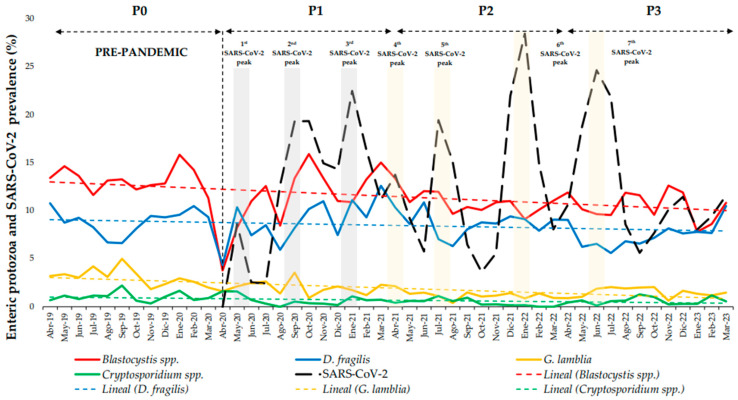
Percentage of prevalence by months for *Blastocystis* spp., *Dientamoeba fragilis*, *Giardia lamblia*, and *Cryptosporidium* spp. during prepandemic (P0, 1 April 2019–31 March 2020) and first (P1, 1 April 2020–31 March 2021), second (P2, 1 April 2021–31 March 2022), and third (P3, 1 April 2022–31 March 2023) pandemic years and SARS-CoV-2 peaks of infection.

During the first three SARS-CoV-2 peaks of infection in Spain [10] (period P1), a direct relationship was observed between the increase in SARS-CoV-2 and EP prevalences. However, from the fourth to the seventh SARS-CoV-2 peaks [10] (periods P2 and P3), there was an inverse relationship with a decrease in the prevalence of EP during the peaks of SARS-CoV-2 infection (Figure 1).

There was no great variation in the median age between the overall EP-positive patients from the different periods, with a maximum difference of ±6 years between periods (Table 1). The overall AI rate showed a statistically significant decrease (*p* < 0.05) between the prepandemic and every pandemic period. The greatest decrease in AI occurred during P1 (Table 2).

### 3.2. Trends in Enteric Protozoa During SARS-CoV-2 Pandemic

There was a linear decrease in the overall number of positive RT-PCR patients with EP between P0 and P3, with the most significant decrease occurring during the first pandemic year (P1) because of the severe disruption of health system services due to the start of the SARS-CoV-2 pandemic (Table 1, Figure 1).

**Figure 2:**
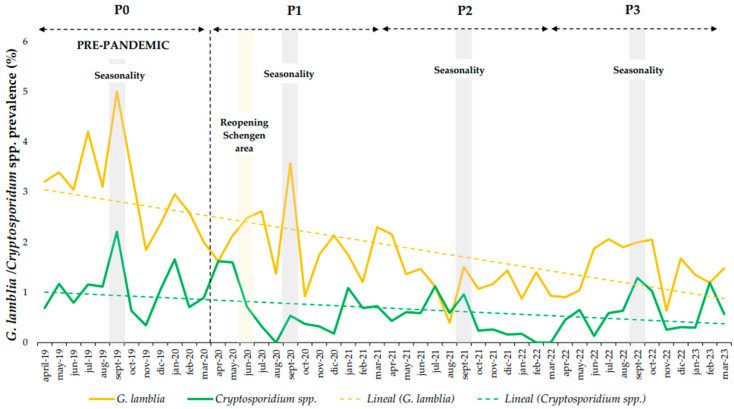
Percentage of prevalence by months for *Giardia lamblia* and *Cryptosporidium* spp. during prepandemic (P0, 1 April 2019–31 March 2020), and first (P1, 1 April 2020–31 March 2021), second (P2, 1 April 2021–31 March 2022), and third (P3, 1 April 2022–31 March 2023) pandemic years with primarily peaks of infection.

Relating to P0, the overall prevalence of EP decreased by 19.4% during P3, 17.7% during P2, and 8.9% during P1. This decrease during the SARS-CoV-2 pandemic was mainly because of the reduction in positive *Blastocystis* spp. and *D. fragilis* RT-PCRs. Both protozoa showed the highest decrease in prevalence during P2–P3 (*p* < 0.05) than during P1, and the lowest AI during P1 (*p* < 0.05). However, during the SARS-CoV-2 pandemic, *G. lamblia* and *Cryptosporidium* spp. showed a decrease in prevalence and AI during P1–P3 (*p* < 0.05), with an increase in cases during P3, and the highest decrease in prevalence and AI during P2 (*p* < 0.05) (Table 1 and Table 2) (Figure 2). Finally, there was a maintenance trend for *E. histolytica-* and *C. cayetanensis*-positive RT-PCRs, with a slight decrease during P1 and P2, although there were no remarkable variations during the study period due to the low number of RT-PCR-positive patients during the four periods (n < 10).

### 3.3. Patients Attending a National Referral Center for Imported Tropical Diseases (NRCITD)

The number of patients attending an NRCITD decreased drastically at the beginning of the SARS-CoV-2 pandemic (i.e., 57.6% fewer medical consultations in P1, 39% fewer in P2, and 24.2% fewer in P3, compared to P0). The number of NRCITD patients screened for EP decreased during the pandemic: during P1, the number of NRCITD patients screened decreased by 70.7% compared to that during P0. During P2, the number of NRCITD patients analyzed showed a slight increase compared to that during the P1 period (with a decrease of 68.9% with respect to P0). This increase was higher during the P3 compared to the P1–P2 periods, but 41.9% fewer patients were analyzed during P3 compared to in P0 (Table 3).

### 3.4. Trends in Enteric Protozoa in NRCITD Patients

**Figure 3:**
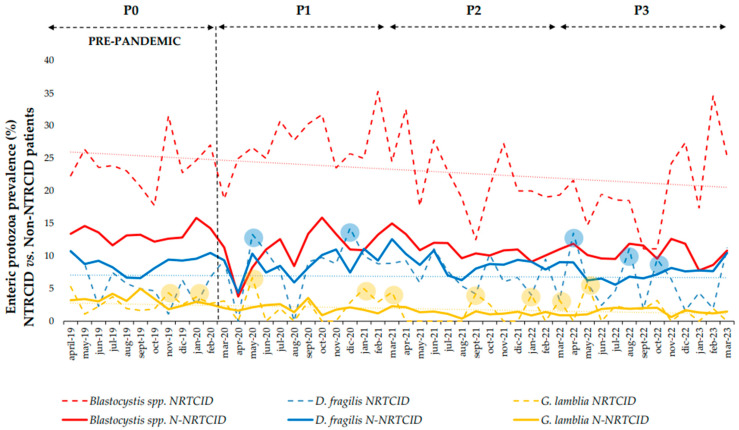
Percentage of prevalence by month for *Blastocystis* spp., *Dientamoeba fragilis*, and *Giardia lamblia* for patients consulted at a National Referral Center for Imported Tropical Diseases (NRCITD) vs. non-NRCITD during prepandemic period (P0, 1 April 2019–31 March 2020) and first (P1, 1 April 2020–31 March 2021), second (P2, 1 April 2021–31 March 2022), and third (P3, 1 April 2022–31 March 2023) pandemic years with the highest peaks of infection rounded off in the NTRCID population.

The subanalysis was performed only for *Blastocystis* spp., *D. fragilis*, and *G. lamblia.* Comparing the monthly prevalences of the NRCITD vs. non-NRCITD patients within each period, there was only a higher prevalence of *Blastocystis* spp., with a statistically significant difference (T–test, *p* < 0.001) for NRCITD patients during the four periods studied (Figure 3).

When comparing the prevalence of the NRCITD patients between P0 and the three pandemic periods, there was an overall increase during P1 (*p* < 0.05) and a decrease in P3 (*p* < 0.05) that was statically significant. The AI again showed the greatest decrease during P2 (Table 3).

*Blastocystis* spp. showed the same trends (*p* < 0.05) as the overall NTRCID results. *D. fragilis* showed a non-statistically significant decrease (*p* > 0.05) in prevalence during P3 in comparison with P0. *G. lamblia* showed a slight increase in prevalence during P1 (*p* > 0.05) with a decrease during P2–P3 (*p* > 0.05) (Table 3). *Cryptosporidium* spp., *C. cayetanensis*, and *E. histolytica* were not analyzed because of the few positive RT-CR results during the four periods analyzed (n < 10). However, the prevalence of *E. histolytica* and *C. cayetanensis* were associated primarily with the NRCITD patients.

## 4. Discussion

Our study provides the impact on the prevalence of, incidence of, and trends in EP in an overall population of a tertiary-care hospital and NRCITD patients during the SARS-CoV-2 pandemic in Spain. This global decrease in EP has been reported in other countries [11,12,13,14,15,16,17]. However, Meena et al. reported an increase in EP during the SARS-CoV-2 pandemic [18].

The greatest reduction in the prevalence of EP, along with the lowest AI, occurred during the P1 period, as expected, due to the strict confinement measures implemented by the Spanish government, the restrictions imposed on the population, and NPIs in place during the SARS-CoV-2 pandemic [19]. Furthermore, the overall prevalence trend decreased during the three pandemic periods. This decrease was also observed for viral or bacterial gastrointestinal infections in this health area [4,5]. The lack of water or food sanitation during the SARS-CoV-2 pandemic did not appear to have a major impact on high-income countries such as Spain in comparison with low-income countries [11,12,13,14,15,16,17].

During the pandemic, two trends were observed between the EP and SARS-CoV-2 prevalences: during P1, there was a directly proportional increase. This may be attributed to SARS-CoV-2 infection also being able to cause gastrointestinal symptoms. Screening of diarrheal samples without suspected EP infection at the beginning of the pandemic may have led to the increased detection of commensal EP during this period. However, this trend reversed during P2 and P3, likely because of a better understanding of the physiopathology of SARS-CoV-2 and its potential to cause gastrointestinal symptoms. Moreover, the restricted NPIs implemented during P1 could have had a higher impact on the EP prevalence during P2 and P3. In terms of the seasonal predominance, there is a higher incidence of EP during spring/summer because EP infections are associated with fecal–oral transmission through contaminated water or food consumption, and recreational water sports, among others [20,21]. Typical increases were observed in these months during the prepandemic and pandemic periods.

Due to SARS-CoV-2 measures, there was an almost complete reduction in international travel [2] and the number of NRCITD patients attending the center and screened for EP was reduced. During P2, fewer EP cases were detected among the NRCITD population despite an increase in the number of patients attending when compared to P1. This could be because of the three SARS-CoV-2 peaks in infection during this period, including the biggest SARS-CoV-2 peak in December 2021 (one of the largest worldwide) [10]. This may have led to a reduction in international travel due to border closures as well as the intense application of NPIs by the population during travel. Moreover, Spain could have been implementing stricter NPIs during the pandemic than other countries, with some of them mandatory until as late as 2023. In addition, because of the pandemic, the use of immunocompromising drug regimens for the treatment of SARS-CoV-2 infection (i.e., prolongation of treatment days at high doses) and, similarly, the increase in intensive care unit admissions, could have resulted in a larger population group with favorable characteristics for symptomatic EP infections, which would not have occurred in the absence of the SARS-CoV-2 pandemic.

*Blastocystis* spp. was the most commonly detected EP during the four periods in the NRCITD and non-NRCITD patients. There was a decreasing trend in the prevalence of NRCITD and non-NRCITD patients, with a major decrease during P2–P3. However, the highest AI decrease was during the P1 period. The maximum prevalences coincided with the SARS-CoV-2 peaks in infection (Figure 1). NPIs favored the reduction in *Blastocystis* spp. transmission, primarily during the first year of the pandemic. However, the prevalence was probably maintained during P1 because of the detection of *Blastocystis* spp. in SARS-CoV-2 patients. The detection of *Blastocystis* spp. does not imply illness [8,22,23,24] (∼60% of populations screened are colonized by *Blastocystis* spp. [8]). However, their detection in stool is related to the consumption of contaminated water/food and poor hygiene habits [8]. A *Blastocystis* spp. prevalence decrease was reported in other high-income countries, such as Saudi Arabia, during SARS-CoV-2 pandemic [12]. However, in low-income countries, the inadequate water treatment and healthcare crisis during the pandemic may have facilitated an increase in the *Blastocystis* spp. prevalence, such as in Lebanon or India [14,18]. In our study, we observed a higher prevalence of *Blastocystis* spp. in the NRCITD vs. the non-NRCITD patients. This could be explained because the NRCITD patient profile is related to a higher frequency of travel and exposure to an unhygienic environment facilitating *Blastocystis* spp. transmission [25]. Gefen-Halevi *et al.* reported a higher *Blastocystis* spp. prevalence compared to classical traveler-associated pathogens such as *E. histolytica* or *G. lamblia* even during the prepandemic period [25]. This change could be due to the introduction of molecular techniques in clinical microbiology laboratories with higher sensitivity compared to classical microscopy [26]. However, in our study, the patients were screened using the same diagnostic RT-PCR assay during the four periods.

*D. fragilis* was the second most common EP detected among the NRCITD and non-NRCITD patients. Similarly to *Blastocystis* spp., there has been a decrease in the prevalence and AI after the onset of the pandemic, with a lower prevalence observed during P2–P3. However, during P1, *D. fragilis* showed a slight increase in prevalence compared to that in P0. This could be explained because performing EP RT-PCRs during the first SARS-CoV-2 peaks (P1) may have led to the detection of a higher number of patients colonized with *D. fragilis* (∼68% of populations screened are colonized by *D. fragilis* [8]) primarily in high-income countries, such as Spain. However, *D. fragilis* detection does not imply illness either [8,27]. The monthly increases/decreases in *D. fragilis* and *Blastocystis* spp. had the same profile trend during the period studied (Figure 1). This could lead to the conclusion that the transmission of both EP could be common [28]. In relation to the group of NRCITD patients, during holiday periods, there was a higher prevalence of *D. fragilis* than that in non-NRCITD patients returning from low-income countries (Figure 3) [25]. Performing RT-PCRs in NRCITD patients with suspected classical EP (i.e., *G. lamblia*) may have led to the higher detection of *D. fragilis* in this group.

*G. lamblia* was the third most prevalent EP in the NRCITD and non-NRCITD patients. A decrease in the prevalence and AI was shown during the pandemic, with the highest decrease in both during P2. This decrease could be explained by travel during P2 to endemic areas being reduced. Spanish national giardiasis data have reported the same general decrease since 2020 [29]. During P1, NRCITD patients showed a slight increase in prevalence and AI despite a ∼58% decrease in the number of NRCITD patients screened during the P1 period. This could be explained by *G. lamblia* being an EP directly associated with traveler/VFR patients going to/coming from low-income countries during the summer months (Figure 2). The lack of water sanitation could have been affected during the SARS-CoV-2 pandemic in these areas. Moreover, *G. lamblia* cysts are resistant to chlorine favoring their transmission. On June 2020 (P1), an increase in the *G. lamblia* prevalence coincided with the reopening of the Schengen area and an increase in international trips [30]. In the September of each period, there was a prevalence increase in accordance with holiday periods. This was higher in September 2020, probably because it was the first holiday period after confinement, allowing international travel from Spain [31] (Figure 2).

*Cryptosporidium* spp. infection showed a low decrease in prevalence but a great decrease in AI during the SARS-CoV-2 pandemic, with the highest decrease during P2, similar to *G. lamblia*, because of NPI implementation. Spanish national cryptosporidiosis data have also reported a decrease since 2020 [32]. *Cryptosporidium* spp. infections can cause epidemic outbreaks in immunosuppressed patients and children [33]. In addition, zoonotic transmission plays an important role in the transmission of this EP [34]. In addition, *Cryptosporidium* spp. oocysts are resistant to water chlorination, which facilitates transmission and outbreaks despite the disinfection of drinking and recreational water [33]. However, during the study period, no *Cryptosporidium* spp. outbreaks were reported in Spain that could explain the peaks in detection [32,35]. Seasonal peaks of cryptosporidiosis occurred during the summer months and are probably associated with water consumption and recreational aquatic activities (reopening in July 2020) that are common during the summer [33]. During the four periods studied, there was a higher number of cases in the pediatric population. The zoonotic transmission of *Cryptosporidium* spp. may occur between children population and pets [34]. Contact between children and pets during the pandemic could also justify the maintenance of the prevalence of cryptosporidiosis in this population. In addition, the maintenance of *Cryptosporidium* spp. detection may be explained by the fact that cryptosporidiosis infection in the pediatric population includes associated symptoms such as fever, vomiting, and abdominal pain, with a propensity for prolonged duration of acute diarrhea [36]. During the pandemic, children with acute diarrhea (i.e., *Cryptosporidium* spp.) probably continued to attend health services. This characteristic was also established in our health area for some bacterial gastrointestinal infections such as *Campylobacter* spp., *Salmonella* spp., and *Y. enterocolitica* during the pandemic [5]. Moreover, the NPIs adopted during the SARS-CoV-2 pandemic in Spain could have interrupted person-to-person transmission of *Cryptosporidium* spp. Once these NPIs were lifted, the empty niche left by some subtypes could have been filled by opportunistic *Cryptosporidium* spp. such as the IfA12G1R5 subtype, whose incidence has expanded in Spain in the recent years [37].

*E. histolytica* and *C. cayetanensis* were the EP with the lowest prevalence. These EP are most often associated with gastrointestinal infections in low-income countries. An increase in these EP has been reported in developing areas [12,14,38]. Moreover, *E. histolytica* and *C. cayetanensis* are associated with chronic illness in returning travelers [39,40]. During P1 and P2, there was a slight decrease or stabilization in the number of *E. histolytica* cases reported also in other countries [12,14]. However, in low-income countries such as Egypt, Yemen, or the Arab Gulf area, an increase in *E. histolytica* cases was reported due to unhygienic practices and migratory movements [40,41]. *C. cayetanensis* showed an increase in cases during P3 associated with NRCITD patients, probably because of the lifting of the international travel restriction 

As limitations, firstly, this study was carried out at a single center, so it was not possible to determine global trends at the national level. Secondly, although the samples were sent after medical suspicion of gastrointestinal infection by protozoa, we cannot rule out the detection of colonizing EP rather than gastrointestinal infection. In addition, a global screening study of every patient from the HULP for EP detection was not performed to calculate the percentage of patients colonized. Third, no sequencing of the strains was performed to study possible outbreaks or changes in genomic subtypes (i.e., *Cryptosporidium* spp.). Future epidemiological studies should use genomic sequencing to analyze possible changes in EP transmission patterns. Finally, we did not study the severity of EP as a function of the patient profile (NRCITD vs. non-NRCITD patients), the rate of EP coinfections with viral or bacterial gastrointestinal illnesses, or the prevalence of EP in SARS-CoV-2 vs. non-SARS-CoV-2 patients. Further studies should be conducted to associate certain EP with viral or bacterial gastrointestinal diseases, and to associate certain EP causing gastrointestinal symptoms in SARS-CoV-2 patient cohorts.

## 5. Conclusions

The SARS-CoV-2 pandemic led to a decrease in the prevalence and AI of EP caused by *Blastocystis* spp., *D. fragilis*, *G. lamblia*, and *Cryptosporidium* spp. Prepandemic levels were not reached in any pandemic period. *Cyclospora cayetanensis* and *E. histolytica* remained at a very low prevalence during the pandemic, following the trend seen in our study setting.

The EP with a mainly colonizing role such as *Blastocystis* spp. and *D. fragilis* showed a lower decrease in prevalence during the first pandemic year (P1), which may have been due to the molecular detection of these EP in patients with diarrhea during SARS-CoV-2 infection, which could have been mistaken for EP symptoms. The NTRCID patients showed a higher prevalence of *Blastocystis* spp. than the non-NTRCID patients during every period studied.

Protozoa with a pathogenic role such as *G. lamblia* and *Cryptosporidium* spp. showed the lowest prevalence and AI during the second pandemic year (P2), probably due to the decrease in tourism, migration, and traveler movements caused by the SARS-CoV-2 pandemic.

The implementation of NPIs had a greater impact, with a higher decrease in the prevalence and AI of viral gastrointestinal illnesses than EP. The reduction was more comparable to the decrease in bacterial gastrointestinal illnesses. This may be mainly due to the different transmission mechanisms, with fecal–oral transmission being the most frequent route.

The prevalence of EP has shown great variability among pathogens during the SARS-CoV-2 pandemic across different countries [12,14]. This variation can be attributed to sanitary conditions and hygiene practices, water purification systems, and country-specific migration and traveler movement. In addition, parasitological diagnosis using classical microscopic techniques and molecular methods may bias the comparison of results.

## Figures and Tables

**Table 1:** Median age (IQR), absolute number of patients with positive RT-PCRs for each enteric protozoa, and prevalence (PR,%) during prepandemic period (P0), first pandemic year (P1), second pandemic year (P2), and third pandemic year (P3) for overall population. Fisher’s test was performed for compare prevalences between periods (*p*).

	P0 (n_o_ = 9686)			P1 (n_o_ = 5953)			P2 (n_o_ = 7749)			P3 (n_o_ = 8599)			*P*		
Enteric Protozoa	Median Age (IQR)	Positive RT-PCRs (n)	PR% (95% CI)	Median Age (IQR)	Positive RT-PCRs (n)	PR% (95% CI)	Median Age (IQR)	Positive RT-PCRs (n)	PR% (95% CI)	Median Age (IQR)	Positive RT-PCRs (n)	PR% (95% CI)	*p* P0 vs. P1	*p* P0 vs. P2	*p* P0 vs. P3
*Blastocystis* spp.	31 (10–46)	1172	12.1 (11.5–12.8)	29.9 (10–47)	672	11.3 (10.5–12.1)	33.4 (11–51)	801	10.3 (9.7–11)	34 (11–51)	919	10.7 (10–11.4)	0.1	<0.05	<0.05
*D. fragilis*	18.1 (5.2–30)	819	8.5 (7.9–9)	18.7 (6–31)	524	8.8 (8.1–9.5)	18.8 (5–25)	651	8.4 (7.8–9)	18.7 (6–31)	621	7.2 (6.7–7.8)	0.4	0.9	<0.05
*G. lamblia*	25 (5–40)	300	3.1 (2.7–3.5)	18.7 (5–31)	111	1.8 (1.5–2.2)	23.5 (5–46)	100	1.3 (1–1.6)	23.4 (4–38)	118	1.4 (1.1–1.6)	<0.05	<0.05	<0.05
*Cryptosporidium* spp.	10.8 (2–12.2)	104	1.1 (0.9–1.3)	11.9 (3–14)	37	0.6 (0.4–0.9)	15.5 (3.5–27.5)	30	0.4 (0.3–0.5)	10.4 (2–11)	57	0.7 (0.5–0.9)	<0.05	<0.05	<0.05
*E. histolytica*	43.2 (34–50)	5	N/A	N/A	1	N/A	N/A	0	N/A	28.3 (16–40)	4	N/A	N/A	N/A	N/A
*C. cayetanensis*	52.5 (52–53)	2	N/A	N/A	0	N/A	N/A	1	N/A	50 (47–56)	6	N/A	N/A	N/A	N/A
Global	30.1 (19.8–40)	2402	24.8 (23.9–25.6)	19.8 (14–27)	1345	22.6 (21.5–23.7)	22.8 (16–31)	1583	20.4 (19.5–21.3)	27.5 (16–38)	1725	20 (19.2–20.9)	<0.05	<0.05	<0.05

IQR, interquartile range; 95% CI, 95% confidence interval; N/A: not applicable. n_o_: overall population screened for enteric protozoa (EP) using RT-PCR in each period.

**Table 2:** Rate of absolute incidence (AI)* of overall patients with positive RT-PCRs for enteric protozoa per 100,000 population during prepandemic period (P0), first (P1), second (P2), and third pandemic years (P3).

Enteric Protozoa	P0 (n = 531,371)	P1 (n = 536,448)	P2 (n = 528,922)	P3 (n = 531,836)	*p* P0 vs. P1	*p* P0 vs. P2	*p* P0 vs. P3
*Blastocystis* spp.	220.6	125.3	151.4	172.8	<0.05	<0.05	<0.05
*D. fragilis*	154.1	97.7	123.1	116.8	<0.05	0.06	<0.05
*G. lamblia*	56.4	20.7	18.9	22.2	<0.05	<0.05	<0.05
*Cryptosporidium* spp.	19.6	6.9	5.7	10.7	<0.05	<0.05	0.1
*E. histolytica*	0.9	0.2	N/A	0.75	0.5	N/A	0.9
*C. cayetanensis*	0.4	N/A	0.2	1.1	N/A	0.8	0.6
Global	452	250.7	301.4	324.3	<0.05	<0.05	<0.05

(AI)*: number of total positive patients for each enteric protozoa infection per 100,000 population divided between the overall population of each period studied. n: hospital population to which the Hospital Universitario La Paz (HULP) provided healthcare in each period.

**Table 3:** Comparison of prevalence (PR,%) and rate of absolute incidence (AI*) of patients attending (NRCITDp) and not attending (N-NRCITDp) a National Referral Center for Imported Tropical Diseases (NRCITD) during prepandemic period (P0), first pandemic year (P1), second pandemic year (P2), and third pandemic year (P3). Fisher’s test has been performed to compare prevalences between periods (*p*).

	**P0**			**P1**			**P2**			**P3**			***p***		
	Population Screened (N-NRCITDp: n = 8988 NRCITDp: n = 698)		Patients Attending NRCITD (n = 1777)	Population Screened (N-NRCITDp: n = 5749 NRCITDp: n = 204)		Patients Attending NRCITD (n = 753)	Population Screened (N-NRCITDp: n = 7532 NRCITDp: n = 217)		Patients Attending NRCITD (n = 1084)	Population Screened (N-NRCITDp: n = 8194 NRCITDp: n = 405)		Patients Attending NRCITD (n = 1346)	PR% (NRCITD)		
Enteric Protozoa	Positive RT-PCR N-NRCITDp (PR%)	Positive RT-PCR NRCITDp (PR%)	AI NRCITDp	Positive RT-PCR N-NRCITDp (PR%)	Positive RT-PCR NRCITDp (PR%)	AI NRCITDp	Positive RT-PCR N-NRCITDp (PR%)	Positive RT-PCR NRCITD (PR%)	AI NRCITDp	Positive RT-PCR N-NRCITD (PR%)	Positive RT-PCR NRCITD (PR%)	AI NRCITDp	*p* P0 vs. P1 NRCITD	*p* P0 vs. P2 NRCITD	*p* P0 vs. P3 NRCITD
*Blastocystis* spp.	940 (10.4)	232 (33.2)	13,055.7	580 (10.1)	92 (45.1)	12,217.8	734 (9.7)	67 (30.9)	6180.8	811 (9.9)	108 (26.6)	8023.8	<0.05	0.5	<0.05
*D. fragilis*	761 (8.5)	58 (8.3)	3263.9	496 (8.6)	28 (13.7)	3718.4	631 (8.4)	20 (9.2)	1845	589 (7.2)	32 (7.9)	2377.4	<0.05	0.7	0.8
*G. lamblia*	275 (3.1)	25 (3.6)	1406.9	103 (1.8)	8 (3.9)	1062.4	96 (1.3)	4 (1.8)	369	108 (1.3)	10 (2.5)	742.9	0.8	0.2	0.3
Global	1976 (22)	315 (45.1)	17,726.5	1179 (20.5)	128 (62.7)	16,998.7	1461 (19.4)	91 (41.9)	8394.8	1508 (18.4)	150 (37)	11,144.3	<0.05	0.4	<0.05

(AI)*: number of total NRCITD positive patients for each enteric protozoa per 100,000 population divided between the NRCITD population attending the NRCITD unit in each period.

## Data Availability

No new data were created or analyzed in this study. Data sharing is not applicable to this article.
